# Designs and methods for implementation research: Advancing the mission of the CTSA program

**DOI:** 10.1017/cts.2020.16

**Published:** 2020-03-04

**Authors:** Soohyun Hwang, Sarah A. Birken, Cathy L. Melvin, Catherine L. Rohweder, Justin D. Smith

**Affiliations:** 1 Department of Health Policy and Management, UNC Gillings School of Global Public Health, University of North Carolina at Chapel Hill, Chapel Hill, NC, USA; 2 Department of Public Health Sciences, Medical University of South Carolina, Charleston, SC, USA; 3 UNC Center for Health Promotion and Disease Prevention, University of North Carolina at Chapel Hill, Chapel Hill, NC, USA; 4 Department of Psychiatry and Behavioral Sciences, Northwestern University Feinberg School of Medicine, Chicago, IL, USA

**Keywords:** Experimental, implementation research, quasi-experimental, trial designs

## Abstract

**Introduction:**

The US National Institutes of Health (NIH) established the Clinical and Translational Science Award (CTSA) program in response to the challenges of translating biomedical and behavioral interventions from discovery to real-world use. To address the challenge of translating evidence-based interventions (EBIs) into practice, the field of implementation science has emerged as a distinct discipline. With the distinction between EBI effectiveness research and implementation research comes differences in study design and methodology, shifting focus from clinical outcomes to the systems that support adoption and delivery of EBIs with fidelity.

**Methods:**

Implementation research designs share many of the foundational elements and assumptions of efficacy/effectiveness research. Designs and methods that are currently applied in implementation research include experimental, quasi-experimental, observational, hybrid effectiveness–implementation, simulation modeling, and configurational comparative methods.

**Results:**

Examples of specific research designs and methods illustrate their use in implementation science. We propose that the CTSA program takes advantage of the momentum of the field's capacity building in three ways: 1) integrate state-of-the-science implementation methods and designs into its existing body of research; 2) position itself at the forefront of advancing the science of implementation science by collaborating with other NIH institutes that share the goal of advancing implementation science; and 3) provide adequate training in implementation science.

**Conclusions:**

As implementation methodologies mature, both implementation science and the CTSA program would greatly benefit from cross-fertilizing expertise and shared infrastructures that aim to advance healthcare in the USA and around the world.

## Background

### Implementation Research: Definition and Aims

The US National Institutes of Health (NIH) established the Clinical and Translational Science Award (CTSA) program in response to the challenges of translating biomedical and behavioral interventions from discovery to real-world use [1]. By the time the CTSA program was established, hundreds of millions of NIH dollars had been spent on developing evidence to influence a wide swath of clinical and preventive interventions for improving patient-level outcomes (e.g., observable and patient-reported symptoms, functioning, and biological markers). This emphasis on “The 7 Ps”: pills, programs, practices, principles, products, policies, and procedures [2] resulted in little to show in terms of improved health at the population level. When the CTSA program was first created, comparative effectiveness research was viewed as an important approach for moving the results of efficacy and effectiveness studies into practice [3]. By comparing multiple evidence-based interventions (EBIs), clinicians and public health practitioners would be armed with information regarding which treatments and interventions to pursue for specific populations. However, establishing the best available EBI among multiple alternatives only closes the research-to-practice-gap by a small margin. How to actually “make it work” (i.e., implementation) in an expeditious and cost-effective manner remains largely uninformed by traditional comparative effectiveness research approaches. The need for implementation research was discussed in the 2010 publication of “Training and Career Development for Comparative Effectiveness Research Workforce Development” as a necessary means of ensuring that comparative effectiveness research findings are integrated into practice [3]. This translation has not yet been fully realized within the CTSA program.

According to the NIH, implementation research is “the scientific study of the use of strategies to adopt and integrate evidence-based health interventions into clinical and community settings in order to improve patient outcomes and benefit population health. Implementation research seeks to understand the behavior of healthcare professionals and support staff, healthcare organizations, healthcare consumers and family members, and policymakers in context as key influences on the adoption, implementation and sustainability of evidence-based interventions and guidelines [4].” In contrast to effectiveness research, which seeks to assess the influence of interventions on patient outcomes, implementation research evaluates outcomes such as rates of EBI adoption, reach, acceptability, fidelity, cost, and sustainment [5]. The objective of implementation research is to identify the behaviors, strategies, and characteristics of multiple levels of the healthcare system that support the use of EBIs to improve patient and community health outcomes, to better address health disparities [6].

With the distinction between EBI effectiveness research and implementation research comes differences in study design and methodology. This article describes designs and methods that are currently applied in implementation research. We begin by defining common terms, describing the goals, and presenting some overarching considerations and challenges for designing implementation research studies. We then describe experimental, quasi-experimental, observational, effectiveness–implementation “hybrid,” and simulation modeling designs and offer examples of each. We conclude with recommendations for how the CTSA program can build capacity for implementation research to advance its mission of reducing the lag from discovery to patient and population benefit [7].

### Definition of Terms

In this article, we often use “implementation” as shorthand for a multitude of processes and outcomes of interest in the field: diffusion, dissemination, adoption, adaptation, tailoring, implementation, scale-up, sustainment, etc. We use the term “implementation science” to refer to the field of study and “implementation research” in reference to the act of studying implementation. We define “design” as the planned set of procedures to: (a) select subjects for study; (b) assign subjects to (or observe their natural) conditions; and (c) assess before, during, and after assignment in the conduct of the study. With many resources for measurement and evaluation of implementation research trials in the literature [8,9], we focus on the selection and assignment of subjects within the design for the purposes of drawing conclusions about the effects of implementation strategies [10,11]. The goals of implementation research are multifaceted and largely fall within two broad categories: (1) examining the implementation of EBIs in communities or service delivery systems; and (2) evaluating the impact of strategies to improve implementation. The approaches and techniques by which healthcare providers and healthcare systems more generally implement EBIs are via “implementation strategies.” Strategies may target one or more levels within a community or healthcare delivery system (e.g., clinicians, administrators, teams, organizations, and the external environment) and can be used individually or packaged to form multicomponent strategies. Some implementation studies are designed to test, evaluate, or observe the impact of one or more implementation strategies. Others seek to understand implementation context, determinants, barriers, and facilitators that will inform the study design [12].

## Characteristics of Implementation Research Designs

### Study Design

Study design refers to the overall strategy chosen for integrating different aspects of a study in a coherent and logical way to address the research questions. Implementation research designs share many of the foundational elements and assumptions of efficacy research. In many experimental and quasi-experimental implementation research studies, the independent variable of interest is an implementation strategy; in other implementation research studies, variables of interest relate to the implementation context or process. Much like an EBI in a traditional clinical trial, the construct must be well-defined, particularly when conducting an experimental study, a topic we will explore in later sections. Three broad types of study designs for implementation research are experimental/quasi-experimental, observational, and simulation. The basic difference among these types is that experimental and quasi-experimental designs feature a well-defined, investigator-manipulated, or controlled condition (often an implementation strategy) that is hypothesized to effect desired outcomes, whereas observational studies are meant to understand implementation strategies, contexts, or processes. Of note, quasi-experiments apply statistical methods to data from quasi-experimental designs to approximate what, from a scientific perspective, would ideally be achieved with random assignment. Whereas quasi-experiments attempt to predict relationships among constructs, observational studies seek to describe phenomena. Simulation may feature experimental or observational design characteristics using synthetic (not observed) data. Table 1 provides a summary of the definition and uses of specific research designs covered in this article along with references to published studies illustrating their use in implementation science literatures.

**Table 1.** Design types, definitions, uses, and examples from implementation science

Design types	Definitions	Uses	Examples from implementation science
Experimental design			
Between-site design	This design compares processes and output among sites having different exposures	Allows investigators to compare processes and output among sites that have different exposures	Ayieko *et al.* [13] Finch *et al.* [14] Kilbourne *et al.* [15]
Within- and between-site design	The comparisons can be made with crossover designs where sites begin in one implementation condition and move to another	Receiving the new implementation strategy, or when it is unethical to withhold a new implementation strategy throughout the study	Smith and Hasan [16] Fuller *et al.* [17]
Quasi-experimental design			
Within-site design	This design examines changes over time within one or more sites exposed to the same dissemination or implementation strategy	These single-site or single-unit (practitioner, clinical team, healthcare system, and community) designs are most commonly compared to their own prior performance	Smith *et al.* [18] Smith *et al.* [19] Taljaard *et al.* [20] Yelland *et al.* [21]
Observational			
Observational (descriptive)	Describes outcomes of interest and their antecedents in their natural context	Useful for evaluating the real-world applicability of evidence	Harrison *et al.* [22] Salanitro *et al.* [23]
Other designs/methods			
Configurational comparative methods	Combine within-case analysis and logic-based cross-case analysis to identify determinants of outcomes such as implementation	Useful for identifying multiple possible combinations of intervention components and implementation and context characteristics that interact to produce outcomes	Kahwati *et al.* [24] Breuer *et al.* [25]
Simulation studies	A method for simulating the behavior of complex systems by describing the entities of a system and the behavioral rules that guide their interactions	Offer a solution for understanding the drivers of implementation and the potential effects of implementation strategies	Zimmerman *et al.* [26] Jenness *et al.* [27]
Hybrid Type 1	Tests a clinical intervention while gathering information on its delivery and/or on its potential for implementation in a real-world situation, with primary emphasis on assessing intervention effectiveness	Offers an ideal opportunity to explore implementation to plan for future implementation	Lane-Fall *et al.* [28] Ma *et al.* [29]
Hybrid Type 2	Simultaneously tests a clinical intervention and an implementation intervention/strategy	Able to assess intervention effectiveness and feasibility and/or potential impact of an implementation strategy receive equal emphasis	Garner *et al.* [30] Smith *et al.* [31]
Hybrid Type 3	Primarily tests an implementation strategy while secondarily collecting data on the clinical intervention and related outcomes	When researchers aim to proceed with implementation studies without completion of the full or at times even a modest portfolio of effectiveness studies beforehand	Bauer *et al.* [32] Kilbourne *et al.* [33]

### Experimental Designs

Experimental design is regarded as the most rigorous approach to show causal relationships and is labeled as the “gold-standard” in research designs with respect to internal validity [34]. Experimental design relies on the random assignment of subjects to the condition of interest; random assignment is intended to uphold the assumption that groups (usually experimental vs. control) are probabilistically equivalent, allowing the researcher to isolate the effect of the intervention on the outcome of interest. In implementation research, the experimental condition is often a specific implementation strategy, and the control condition is most often “implementation as usual.” Brown *et al.* [2] described three broad categories of designs providing within-site, between-site, and within- and between-site comparisons of implementation strategies. *Within-site designs* are discussed in the section on quasi-experimental designs as they generally lack the replicability standard given their focus on one site or unit. It is important to acknowledge that other authors, such as Miller *et al.* [35] and Mazzucca *et al.* [36], have categorized certain designs somewhat differently than we have here.

As research advances through the translational research pipeline (efficacy to effectiveness to dissemination and implementation), study design tends to shift from valuing internal validity (in efficacy trials) to achieving a greater balance between internal and external validity in effectiveness and implementation research. Much in the same way that inclusion criteria for patients are often relaxed in an effectiveness study of an EBI to better represent real-world populations, implementation research includes delivery systems and clinicians or stakeholders that are representative of typical practices or communities that will ultimately implement an EBI. The high degree of heterogeneity in implementation determinants, barriers, and facilitators associated with diverse settings makes isolating the influence of an implementation strategy challenging and is further complicated by nesting of clinicians within practices, hospitals within healthcare systems, regions within states, etc. Thus, the implementation researcher seeks to ensure that any observed effects are attributable to the implementation strategy/ies being investigated and attempts to balance internal and external validity in the design.

#### Between-site designs

In between-site designs, the EBI is held constant across all units to ensure that observed differences are the result of the implementation strategy and not the EBI. Between-site designs allow investigators to compare processes and output among sites that have different exposures. Most commonly the comparison is between an implementation strategy and implementation as usual. Brown and colleagues emphasize that randomization should be at the “level of implementation” in the between-site designs to avoid cross-contamination [2]. Ayieko *et al.* [13] used a between-site design to examine the effect of enhanced audit and feedback (an implementation strategy) on uptake of pneumonia guidelines by clinical teams within Kenyan county hospitals. They performed restricted randomization, which involved retaining balance between treatment and control arms on key covariates including geographic location and monthly pneumonia admissions. The study used random intercept multilevel models to account for any residual imbalances in performance at baseline so that the findings could be attributed to the audit and feedback, the implementation strategy of interest [12].

A variant between-site design is the “head-to-head” or “comparative implementation” trial in which the investigator controls two or more strategies, no strategy is implementation as usual, no site receives all strategies, and results are compared [2]. Finch *et al.* [14] examined the effectiveness of two implementation strategies, performance review and facilitated feedback, in increasing the implementation of healthy eating and physical activity-promoting policies and practices in childcare services in a parallel group randomized controlled trial design. At completion of the intervention period, childcare services that received implementation as usual were also offered resources to use the implementation strategies.

When achieving a large sample size is challenging, researchers may consider matched-pair randomized designs, with fewer units of randomization, or other adaptive designs for randomized trials [37] such as the factorial/fractional factorial [38] or sequential multiple assignment randomized trial (SMART) design. The SMART design allows for building time-varying adaptive implementation strategies (or stepped-care strategies) based on the order in which components are presented and the additive and combined effects of multiple strategies [15]. Kilbourne *et al.* assessed the effectiveness of an adaptive implementation intervention involving three implementation strategies (replicating effective programs [39], coaching, and facilitation) on cognitive behavioral therapy delivery among schools in a clustered SMART design [40]. In the first phase, eligible schools were randomized with equal probability to a single strategy vs. the same strategy combined with another implementation strategy. In subsequent phases, schools were re-randomized with different combinations of implementation strategies based on the assessment of whether potential benefit was derived from a combination of strategies. Similar to the SMART design is the full or fractional factorial design in which units are assigned a priori to different combinations of strategies, and main and lower order effects are tested to determine the additive impact of specific strategies and their interactions [41].

Another between-site design variant, the incomplete block, is useful when two implementation strategies cannot or were not initially intended to be directly compared. The incomplete block design allows for an indirect comparison of the two strategies by drawing from two independent samples of units, one in which sites are randomized to either strategy A or implementation as usual, and the other in which sites are randomized to strategy B or implementation as usual [42]. The two samples are completely independent and can occur either in parallel or in sequence, and statistical tests are performed for indirect comparison of the impacts of the two strategies “as if” they were directly compared. This requires a single EBI to be implemented and some degree of homogeneity across both of the groups. The incomplete block design is useful when it is not possible to test both strategies in a single study, or when a prior or concurrent study can be leveraged to compare two strategies.

Although the examples of between-site designs are randomized at the site- and organization-level, smaller units within each organization such as the team or clinician may also be randomized to an intervention [2]. Smith, Stormshak, & Kavanagh [18] present the results of a study in which clinicians were randomized to receive training or not, and their assigned families were randomized to receive the EBI or usual services. Effectiveness (family functioning and child behaviors) and implementation outcomes (adoption and fidelity) were evaluated after the 2-year period of intervention delivery.

#### Within- and between-site designs

This design involves crossovers where units begin in one condition and move to another (within-site element), which is repeated across units (or clusters of units) with staggered crossover points (between-site element). This broad class of designs has been referred to as “roll-out” designs [43] and dynamic wait-list designs [44]. We use the term “roll-out” to describe within- and between-site designs. The defining characteristic of roll-out designs is the assignment of all units in the study to the time when the implementation strategy will begin (i.e., the crossover). Assignments within roll-out designs can either be random, non-random, or quasi-random. In the context of implementation research, the roll-out design offers three practical and scientific advantages. First, all units in the trial will eventually receive the implementation strategy. Ensuring that all participating units receive the strategy promotes equity and enables all participants to contribute data. Second, the roll-out design allows the research team and the partner organizations to distribute resources required to administer the implementation strategy over time, rather than having to implement in all sites simultaneously as might be done in another type of multisite design. Third, the design allows researchers to account for the effect of unanticipated confounders (e.g., change in accreditation standards that requires use of the implementation strategy) that can occur during the trial period. For example, if some sites start implementation before an external event occurs, and other sites start afterwards, the impact of the event on the implementation process and resulting outcomes can be measured.

**Fig. 1.**
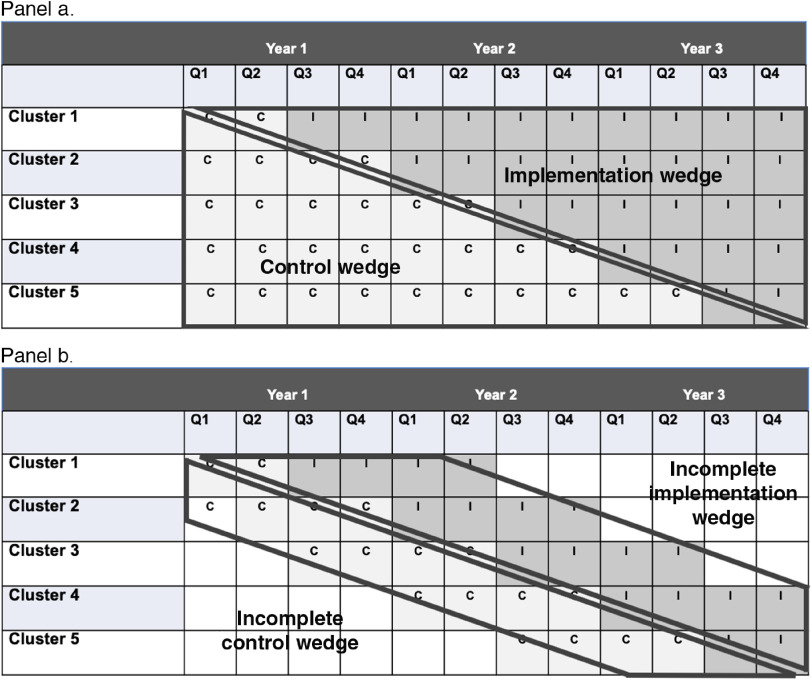
Roll-out designs: the stepped wedge (panel a) and incomplete wedge (panel b).

A common roll-out design is the stepped-wedge. The stepped-wedge is a specific design in which measurement of all units begins simultaneously at T0 and units cross over from one condition (e.g., implementation as usual or usual care) to the experimental implementation strategy condition following a series of “steps” at a predetermined interval (steps refer to the crossover). The result is a “wedge” below the steps of implementation as usual that can be compared to the wedge above the step representing the implementation strategy condition. The stepped-wedge is illustrated in Fig. 1 (panel a).

A variant of this design is the incomplete (or modified) wedge roll-out design (Fig. 1, panel b). The difference from the stepped-wedge is that pre-implementation outcomes measurement begins immediately prior (e.g., 4–6 months) to the step rather than at T0 [16]. Incomplete wedge roll-out designs might be preferred to the traditional stepped-wedge design because there is less burden on participating sites to collect data for long periods and it allows researchers the option of staged enrollment in the trial if needed to achieve the full target sample in a way that does not threaten the study protocol. In this latter situation, randomization would occur in as few stages as possible to maintain balance and a variable for stage of enrollment would be included in all analyses to account for any differences in early vs. later enrollees. Last, the unit of randomization can be single units, clusters, or repeated, matched pairs [45]. Smith and Hasan [16] provide a case example of an incomplete wedge roll-out design in a trial testing the implementation of the Collaborative Care Model for depression management in primary care practices within a large university health system. In that trial, measurement of implementation began 6 months prior to the crossover to implementing the Collaborative Care Model in each primary care practice in a multi-year roll-out.

### Quasi-Experimental Designs

Quasi-experimental designs share experimental design goals of assessing the effect of an intervention on outcomes of interest. Unlike experiments, however, quasi-experiments do not randomly assign participants to intervention and usual care groups. This key distinction limits the internal validity of quasi-experimental designs because differences between groups cannot be attributed exclusively to the intervention. However, when randomization is not possible or desirable for assessing the effectiveness of an implementation strategy or other intervention, quasi-experimental designs are appealing. Internal validity is strengthened when techniques of varying strength are used to bolster internal validity in lieu of randomization, including pre- and post-; interrupted time-series; non-equivalent group; propensity score matching; synthetic control; and regression-discontinuity designs [46].

In the context of implementation research, quasi-experimental designs fall under Brown and colleagues’ broad category of *within-site designs.* These single-site or single-unit (practitioner, clinical team, healthcare system, and community) designs are most commonly compared to their own prior performance. The simplest variant of a within-site study is the post design. This design is relevant when a site or unit has not delivered a service before, and thus, has no baseline or pre-implementation strategy data for comparison. The result of such a study is a single “post” implementation outcome that can only be compared to a criterion metric or the results of published studies. In contrast to a post design where data are only available after an implementation strategy or other intervention is introduced, a pre-post design compares outcomes following the introduction of an implementation strategy to the results from care as usual prior to introducing the implementation strategy.

To increase power and internal validity of within-site studies, interrupted time-series designs can be used [47]. Time-series designs involve multiple observations of the dependent variable (e.g., implementation) before and after the introduction of the implementation strategy, which “disrupts” the time-series data stream. Time-series designs are highly flexible and can involve multiple sites in the multiple baseline and replicated single-case series variants, which increase internal validity through replication of the effect. Examples of interrupted time-series studies exist in implementation research that exemplify their practicality for studying implementation (see Table 1). Limitations of this design in implementation research include the challenge of defining the interruption (i.e., when the implementation began) and that the effects of new implementations are unlikely to be immediate. Therefore, analysis of interrupted time-series in implementation research might favor examining changes in slope between pre-implementation and implementation phases, rather than testing immediate changes in level of the outcome after the interruption.

### Observational Designs

In observational studies, the investigator does not intervene with study participants but instead describes outcomes of interest and their antecedents in their natural context [48]. As such, observational studies may be particularly useful for evaluating the real-world applicability of evidence. Observational designs may use approaches to data collection and analysis that are quantitative [16] (e.g., survey), qualitative [49] (e.g., semi-structured in-depth interviews), or mixed methods [50] (e.g., sequential, convergent analysis of quantitative and qualitative results). Quantitative, qualitative, and mixed methods can be especially helpful in observational studies for systematically assessing implementation contexts and processes.

### Hybrid Designs

With the goal of more rapidly translating evidence into routine practice, Curran *et al.* [51,52] proposed methods for blending: 1) design components of experiments intended to test the effectiveness of clinical interventions and 2) approaches to assessing their implementation. Such hybrid designs provide benefits over pursuing these lines of research independently or sequentially, both of which slow the progress of translation. Curran and colleagues state that effectiveness–implementation hybrid designs have a dual, a priori focus on assessing clinical effectiveness and implementation [51,52]. Hybrids focus on both effectiveness and implementation but do not specify a particular trial design. That is, the aforementioned experimental and observational designs can be used for any of the hybrid types. References to hybrid studies in implementation science are provided in Table 1.

Curran *et al.* describe the conditions under which three different types of hybrid designs should be used, which helps researchers determine the most appropriate type based on whether evidence of effectiveness and implementation exists. Linking clinical effectiveness and implementation research designs may be challenging, as the ideal approaches for each often do not share many design features. Clinical trials typically rely on controlling/ensuring delivery of the clinical intervention (often by using experimental designs) with little attention to implementation processes likely to be relevant to translating the intervention to general practice settings. In contrast, implementation research often focuses on the adoption and uptake of clinical interventions by providers and/or systems of care [53] often with the assumption of clinical effectiveness demonstrated in previous studies. The three hybrid designs are described below.

#### Hybrid Type 1

Hybrid Type 1 tests a clinical intervention while gathering information on its delivery and/or potential for implementation in a real-world context, with primary emphasis on clinical effectiveness. This type of design advocates process evaluations of delivery/implementation during clinical effectiveness trials to collect information that may be valuable in subsequent implementation research studies, answering questions such as: What potential modifications to the clinical intervention could be made to maximize implementation? What are potential barriers and facilitators to implementing this intervention in the “real world”? Hybrid Type 1 designs provide the opportunity to explore implementation and plan for future implementation.

#### Hybrid Type 2

Hybrid Type 2 simultaneously tests a clinical intervention and an implementation intervention/strategy. In contrast to the Hybrid Type 1 design, the Hybrid Type 2 design puts equal emphasis on assessing both intervention effectiveness and feasibility and/or potential impact of an implementation strategy. In a Hybrid Type 2 study, where an implementation intervention/strategy is simultaneously tested to promote uptake of the clinical intervention under study. Type 2 hybrid designs appear less frequently than the other two types due to the resources required.

#### Hybrid Type 3

Hybrid Type 3 primarily tests an implementation strategy while secondarily collecting data on the clinical intervention and related outcomes. This design can be used when researchers aim to proceed with implementation studies without an existing portfolio of effectiveness studies. Examples of these conditions are when: health systems attempt implementation of a clinical intervention without comprehensive clinical effectiveness data; there is strong indirect efficacy or effectiveness data; and potential risks of the intervention are limited. National priorities (e.g., the opioid epidemic) may also drive implementation before effectiveness data are robust.

### Modeling

Implementation research is, by definition, a systems science in that it simultaneously studies the influence of individuals, organizations, and the environment on implementation [54]. The field of systems science is devoted to understanding complex behaviors that are both highly variant and strongly dependent on the behaviors of other parts of the system. Systems science is a challenging field to study using traditional clinical trial methods for various reasons, most notably the complexity involved in the many interactions and dynamics of multiple levels, constant change, and interdependencies. Simulation studies offer a solution for understanding the drivers of implementation and the potential effects of implementation strategies [55]. Modeling typically involves simulating the addition or configuration of one or more specific implementation strategies to determine which path should be taken in the real world, but it can also be used to test the likely effect of implementing one or more EBIs to determine impact for specific populations.

Agent-based modeling (ABM) [56] and participatory systems dynamics modeling (PSDM) [57] have both been used in implementation research to model the behavior of systems and determine the impact of moving certain implementation “levers” in the system. ABM is a method for simulating the behavior of complex systems by describing the entities (called “agents”) of a system and the behavioral rules that guide their interactions [56]. These agents, which can be any element of a system (e.g., clinicians, patients, and stakeholders), interact with each other and the environment to produce emergent, system-level outcomes [58], many of which are formal implementation outcomes. As ABM produces a mechanistic model, researchers are able to identify the implementation drivers that should be leveraged to most effectively achieve the predicted impacts in practice. Whereas ABM has wide ranging applications for implementation science, PSDM is an example of a method for a specific implementation challenge. Zimmerman *et al.* [26] used PSDM to triangulate stakeholder expertise, healthcare data, and modeling simulations to refine an implementation strategy prior to being used in practice. In PSDM, clinic leadership and staff define and evaluate the determinants (e.g., clinician knowledge, implementation leadership, and resources) and mechanisms (e.g., self-efficacy, feasible workflow) that determine local capacity for implementation of an EBI using a visual model. Given local capacity and other factors, simulations predict overall system behavior when the EBI is implemented. The process is iterative and has been used to prepare for large initiatives where testing implementation using standard trial methods was infeasible or undesirable due to the cost and time involved.

### Configurational Comparative Methods

Configurational comparative methods, which are an umbrella term for methods that include but are not limited to qualitative comparative analysis [59], combine within-case analysis and logic-based cross-case analysis to identify determinants of outcomes such as implementation. Configurational comparative methods define causal relationships by identifying INUS conditions: those that are an Insufficient but Necessary part of a condition that is itself Unnecessary but Sufficient for the outcome. Configurational comparative methods may be preferable to standard regression analyses often used in quasi-experiments when the influence of an intervention on an outcome is not easily disentangled from how it is implemented or the context in which it is implemented – i.e., complex interventions. Complex interventions often have interdependent components whose unique contributions to a given outcome can be challenging to isolate. Furthermore, complex interventions are characterized by blurry boundaries among the intervention, its implementation, and the context in which it is implemented [60]. For example, the effectiveness of care plans for cancer survivors in improving care coordination and communication among providers likely depends upon a care plan's content, its delivery, and the functioning of the cancer program in which it is delivered [61]. Configurational comparative methods facilitate identifying multiple possible combinations of intervention components and implementation and context characteristics that interact to produce outcomes. To date, qualitative comparative analysis is the type of configurational comparative methods that has been most frequently applied in implementation research [62]. To identify determinants of medication adherence, Kahwati *et al.* [24] used qualitative comparative analysis to analyze data from 60 studies included in a systematic review. Breuer *et al.* [25] used qualitative comparative analysis to identify determinants of mental health services utilization.

### Relevance and Opportunities for Application in CTSAs

In the early days of the CTSA program, resources allocated to implementation science were most frequently embedded in clinical or effectiveness research studies, and few had robust, standalone implementation science programs [63,64]. As the National Center for Advancing Translational Sciences (NCATS) and other federal and non-federal sources have increased their investment in implementation science capacity, the field has grown dramatically. More CTSAs are developing implementation research programs and incorporating stakeholders more fully in this process, as reflected in the results of the Dolor et al [65] environmental scan. Washington University and the University of California at Los Angeles have documented their efforts to engage practice and community partners, offer professional development opportunities, and provide consultations to investigators both in and outside the field of implementation science [66,67]. The CTSA program could take advantage of this momentum in three ways: integrate state-of-the-science implementation methods into its existing body of research; position itself at the forefront of advancing the science of implementation science by collaborating with other NIH institutes that share the goal of advancing implementation science, such as NCI and NHLBI; and providing training in implementation science.

#### Integrating state-of-the-science implementation methods to CTSAs’ existing bodies of research

Many CTSAs have the expertise to consult with their institution's investigators on the potential role of implementation science in their research. Implementation research consultations involve creating awareness and appropriate use of specific study designs and methods that match investigators’ needs and result in meaningful findings for real-world clinical and policy environments. As described by Glasgow and Chambers, these include rapid, adaptive, and convergent methods that consider contextual and systems perspectives and are pragmatic in their approach [68]. They state that “CTSA grantees, among others, are in a position to lead such a change in perspective and methods, and to evaluate if such changes do in fact result in more rapid, relevant solutions” to pressing public health problems. Through consultation services, CTSAs can encourage the use of implementation science early (e.g., designing for dissemination and implementation [69]) and often, positioning CTSAs – the hub for translation – to fulfill their mission by reducing the lag from discovery to patient and population benefit.

#### Advancing the science of implementation science

The centers funded by the CTSA program are able to conduct large-scale implementation research using the multisite U01 mechanism which requires the involvement of three centers. With the challenges of recruitment, generalizability, and power that are inherent in many implementation trials, the inclusion of three or more CTSAs, ideally representing diversity in region, populations, and healthcare systems, can provide the infrastructure for cutting-edge implementation science. Thus far, there are few examples of this mechanism being used for implementation research. In addition, with the charge of speeding translation of bench and clinical science discoveries to population impact, CTSAs have both the incentive and perspective to conduct implementation research early and consistently in the translational pipeline. As the hybrid design illustrates, there has been a paradigmatic shift away from the sequential translational research pipeline to more innovative methods that reduce the lag between translational steps.

#### Training in implementation science

NIH has funded several formal training programs in implementation science, including the Training Institute in Dissemination and Implementation in Health [70], Implementation Research Institute [71], and Mentored Training in Dissemination and Implementation Research in Cancer [72]. These training programs address the need to gain greater clarity around the implementation research designs described in this article, but the demand for training outpaces available resources. CTSAs could provide an avenue for meeting the needs of the field for training in dissemination and implementation science methods. CTSA faculty with expertise in implementation research could offer implementation research training programs for scholars on many levels using the T32, KL2, K12, TL1, R25, and other mechanisms. Chambers and colleagues have recently noted these capacity-building and training opportunities funded by the NIH [73]. Indeed, given the mission of the CTSA program, they are the ideal setting for implementation research training programs.

## Conclusion

The field of implementation science has established methodologies for understanding the context, strategies, and processes needed to translate EBIs into practice. As they mature alongside one another, both implementation science and the CTSA program would greatly benefit from cross-fertilizing expertise, infrastructure, and aim to advance healthcare in the USA and around the world.
